# Tree diversity across the Minneapolis‐St. Paul Metropolitan Area in relation to climate and social vulnerability

**DOI:** 10.1002/eap.3034

**Published:** 2024-09-22

**Authors:** Adrienne B. Keller, Leslie A. Brandt, Jeannine Cavender‐Bares, Joseph F. Knight, Sarah E. Hobbie

**Affiliations:** ^1^ Department of Ecology, Evolution and Behavior University of Minnesota Saint Paul Minnesota USA; ^2^ College of Forest Resources and Environmental Science Michigan Technological University Houghton Michigan USA; ^3^ Office of Sustainability and Climate United States Forest Service Saint Paul Minnesota USA; ^4^ Department of Organismic and Evolutionary Biology Harvard University Cambridge Massachusetts USA; ^5^ Department of Forest Resources University of Minnesota Saint Paul Minnesota USA; ^6^ Department of Forest Resources and Environmental Conservation Virginia Technological University Blacksburg Virigina USA

**Keywords:** biodiversity, climate change, forest management, urban forest

## Abstract

Urban tree canopy cover is often unequally distributed across cities such that more socially vulnerable neighborhoods often have lower tree canopy cover than less socially vulnerable neighborhoods. However, how the diversity and composition of the urban canopy affect the nature of social‐ecological benefits (and burdens), including the urban forest's vulnerability to climate change, remains underexamined. Here, we synthesize tree inventories developed by multiple organizations and present a species‐specific, geolocated database of more than 600,000 urban trees across the 7‐county Minneapolis‐St. Paul (MSP) metropolitan area in the Upper Midwest of the United States. We find that tree diversity across the MSP is variable yet dominated by a few species (e.g., *Fraxinus pennsylvanica*, *Acer platanoides*, and *Gleditsia triacanthos*), contributing to the vulnerability of the MSP urban forest to future climate change and disturbances. In contrast to tree canopy cover, tree diversity was not well predicted by socioeconomic or demographic factors. However, our analysis identified areas where both climate and social vulnerability are high. Our results add to a growing body of literature emphasizing the importance of considering how complex and interacting social and ecological factors drive urban forest diversity and composition when pursuing management objectives.

## INTRODUCTION

Urban canopies promote myriad ecosystem functions and social‐ecological benefits, but their spatial distribution within urban areas is patchy (Cadenasso et al., 2007; Pickett et al., 2011). Consequently, the local benefits of urban trees, including moderated extreme temperatures (Walker et al., 2023; Ziter et al., 2019), reduced stormwater runoff (Berland et al., 2017), absorption of airborne particulate matter (Escobedo & Nowak, 2009; Nowak et al., 2006), and aesthetic value and recreation opportunities that enhance mental health (Bratman et al., 2015), as well as the potential burdens such as increased allergens and tree fall damage to property, are experienced unequally by urban human communities. For example, urban tree canopy cover frequently correlates with demographic and socioeconomic factors (e.g., wealthier and whiter neighborhoods commonly have greater canopy cover; Anderson et al., 2023; Gerrish & Watkins, 2018; Nowak et al., 2022; Volin et al., 2020; Watkins & Gerrish, 2018).

Beyond total canopy cover, the composition of the urban tree canopy has potential to influence both the local benefits and burdens of the urban forest (Morgenroth et al., 2016) and how resistant and resilient the urban forest will be to environmental change (Grossman et al., 2018; Ordóñez & Duinker, 2014). Compared with research on total canopy cover, much less research has examined patterns of urban tree canopy diversity and how tree biodiversity patterns relate to environmental and social vulnerability. Still, recent work suggests urban tree diversity may also vary spatially according to socioeconomic (Anderson et al., 2023) and racial (Burghardt et al., 2023) factors.

Urban areas may be important reservoirs of biodiversity, helping maintain regional species pools (Alvey, 2006; Knapp et al., 2008). Meanwhile, the concurrent threats of increasing land fragmentation (Haddad et al., 2015), which threatens biodiversity globally, and increasing urbanization (United Nations, 2018) emphasize the importance of explicitly considering the urban environment in efforts to protect and promote biodiversity (Schwarz et al., 2017). The diversity of urban trees, as compared to other taxa, can be more easily and directly managed through intentional plantings. Urban tree diversity can promote biodiversity at other trophic levels (e.g., birds: Paker et al., 2014; Savard et al., 2000; and arthropods: Philpott et al., 2014), indicating that management actions related to urban tree composition can support broader urban biodiversity goals.

Tree diversity and composition can affect an ecosystem's capacity to adapt to climate change and increasing disturbance (Brandt et al., 2021; Janowiak et al., 2021). More diverse tree communities are expected to confer greater resistance (i.e., less change in forest cover following perturbations such as disturbance, extreme climate events, and pests and pathogens), and resilience (i.e., quicker and more complete recovery of forest cover following perturbations) than less diverse tree communities (Isbell et al., 2015). Community composition also determines resistance and resilience, as some species have traits better suited to stressors (e.g., drought) than other species (Gillner et al., 2014), while greater overall diversity increases the likelihood of having species present in the community that are well‐adapted to a given stressor. For example, a greater diversity of tree species can decrease tree disease transmission across the landscape and reduce risk of stand‐replacing disease outbreaks, promoting forest resistance (Dale & Frank, 2017). Greater diversity may also allow forest cover to recover more quickly when some species succumb to disease, promoting resilience. Recent work estimates that 1.4 million street trees across the United States will die from invasive insect exposure between 2020 and 2050, with ash tree (*Fraxinus* spp.) death due to the emerald ash borer (*Agrilus planipennis*, EAB) accounting for 90% of all mortality (Hudgins et al., 2022).

Urban trees species identity can also affect the economic, health, social, and cultural characteristics of urban areas (Roy et al., 2012). For example, large shade tree species can be important for moderating air temperature, while small ornamental trees may be valued for their aesthetic or wildlife benefits. Certain species may not be preferred because of, for example, high allergen output or cultural preferences (Roman et al., 2021). Therefore, characterizing urban tree diversity and composition is critical both for understanding the multifaceted roles that trees play in social‐ecological urban systems and to inform urban forest management plans in the face of stressors such as climate change and pest and pathogen outbreaks.

Many of the ecosystem services and disservices from trees occur at very local scales (e.g., urban heat island mitigation, aesthetic value, and storm damage). At the same time, socioeconomic status and racial and ethnic demographics vary spatially across cities. Consequently, how different residents experience the urban forest depends on local scale patterns of tree canopy cover and composition. Within and across cities, total tree canopy cover commonly increases with wealth (Gerrish & Watkins, 2018) and is lower in neighborhoods with greater percentage of minority residents (Locke et al., 2021; Watkins & Gerrish, 2018). Tree canopy diversity showed similar patterns in Baltimore, USA (Anderson et al., 2023; Burghardt et al., 2023), and prior work suggests that wealth is an important predictor of cultivated plant diversity in residential yards in the Minneapolis‐St. Paul (MSP) metropolitan area (Cavender‐Bares et al., 2020). How general such diversity trends are across other cities remains an open question as the relationship between wealth and plant diversity across urban forests can be complex and nonlinear (Hope et al., 2003), suggesting social‐ecological relationships related to tree cover do not necessarily pertain to tree diversity. Most urban tree studies to date have examined biodiversity patterns across a single city or county or conducted cross‐city comparisons spanning a wide geographic range, while fewer studies have examined multiple jurisdictions within a single metropolitan area. Most urban tree biodiversity studies focus exclusively on street trees (e.g., Anderson et al., 2023), and yet different drivers (e.g., nursery availability and affordability, preferences of residents, and natural regeneration) may influence street trees compared with park trees or other domains such as private trees (Avolio et al., 2018; Cavender‐Bares et al., 2020; Dickinson & Ramalho, 2022). We used the newly established urban MSP Long Term Ecological Research (LTER) program, encompassing seven counties and >100 cities, to explore biodiversity patterns of urban street, recreational parkland, and “other” trees across a highly diverse and dynamic social‐ecological system while minimizing climatic differences.

Our study had three goals: (1) Characterize the composition and taxonomic and phylogenetic diversity of urban trees across the MSP seven‐county metropolitan area (“tree diversity patterns”); (2) assess spatial patterns of tree canopy vulnerability to climate change across the region (“tree canopy climate vulnerability”); and (3) assess spatial relationships between tree canopy diversity and social vulnerability, which refers to how external stressors impact public health (“tree diversity relationships to social vulnerability”). We hypothesized that areas (analyzed at the scale of US census tracts) with greater tree taxonomic and phylogenetic diversity would contribute to lower climate vulnerability because greater diversity will give a lower likelihood that any given highly vulnerable species dominates the canopy. We also hypothesized that tree canopy diversity and social vulnerability would be related, such that more socially vulnerable neighborhoods would tend to have lower canopy diversity because of chronic disinvestment. Finally, we hypothesized that areas of the urban forest predicted to be more vulnerable to climate change due to canopy composition would also be in more socially vulnerable neighborhoods with lower capacity to adapt to the effects of climate change.

## METHODS

### Study area

Our study was conducted in the seven‐county MSP metropolitan area, USA, a region that is ecologically, culturally, and politically diverse and well‐suited to studying how social‐ecological factors relate to urban forest diversity. The area lies at the transition between two major biomes (deciduous forest and tallgrass prairie) with a climate that consists of cold, snowy winters and warm, humid summers. Mean annual temperature is 8.3°C and mean annual precipitation is 800 mm (period 1991–2020), 15%–20% of which falls as snow (Palecki et al., 2021). Due to climate change, temperatures are rising in the region and heavy precipitation events are becoming more common, particularly in the winter and spring (Minnesota Department of Natural Resources, 2023; Morgenroth et al., 2016). Intensified urban heat island effects (Smoliak et al., 2015) along with warmer and wetter winters highlight the important challenge of managing for climate‐resilient and ‐resistant urban forests across MSP.

MSP spans 7711 km^2^ with an average population density in 2020 of 1713 people/km^2^ (range across census tracts = 0–17,359/km^2^) and has a total population of more than three million people. Located on the traditional and contemporary Dakota lands with strong ties to the Ojibwe, MSP is currently composed of 26% Black, Indigenous, and other People of Color (BIPOC; Minnesota Compass, 2023), compared with an average of ~9% BIPOC residents across the major metropolitan areas in the United States (Frey, 2022). In 2020, average median household income was $87,737 (range across census tracts = $14,748–$250,001), ranking MSP with the seventh highest median household income of the major metropolitan areas in the country. Percent of owner‐occupied housing averaged 69% (range across census tracts = 0%–100%). These demographic and socioeconomic data were estimated at the scale of a census tract from the U.S. Census 5‐year American Community Survey (ACS) 2020.

### Tree diversity patterns

To examine fine‐scale patterns of tree taxonomic and phylogenetic diversity across MSP, we first compiled a spatially explicit, species‐specific database of urban trees for the region. We solicited existing georeferenced tree inventories from all municipalities, counties, park systems, and relevant non‐profit organizations in the region for which we found contact information. This resulted in inventories from 35 municipalities, one county, one park system, and three nonprofit organizations, and two datasets from prior academic research efforts. The spatial and temporal scope of the inventories varied; for example, the inventories from some municipalities included data from a subset of only street trees from one timepoint, whereas other municipal inventories were continuously updated datasets with spatially comprehensive data for street trees in addition to some trees in parks and private lands. No inventory was fully comprehensive of all trees on public and private property in a given area. Minimum criteria for including data in the database were that trees were georeferenced and identified to genus, with most trees identified to species. Our full dataset covered 83% of census tracts across the MSP, and within Minneapolis, tree diversity data strongly related to canopy cover. This suggests there was not a strong spatial bias to our dataset, particularly for Minneapolis, yet we cannot rule out all biases related to where we had data coverage. Although the timestamp on each data point was not always explicit, metadata indicated that all data could be assumed to have been collected between 2013 and 2022, with most data collected between 2020 and 2022. Recognizing that the inclusion of potentially outdated inventories meant that not all trees in our database were still present at the time of data analysis, our database most accurately provides insight into species‐specific urban forest planting rather than up‐to‐date data on current tree forest diversity. However, inventory metadata and conversations with natural resource managers indicated that most trees were present at time of data analysis.

Individual inventories were combined into one uniform database. Species names were manually resolved to the accepted scientific name according to the Integrated Taxonomic Information System (ITIS; https://www.itis.gov/); the high variability in (mis)spellings and use of common names required manual checking of all names in lieu of an automated script. Hybrid species were prevalent in the database and were treated as a unique species, given that hybrids can have distinct traits from their parents (Rieseberg et al., 1993). In approximately 10% of cases, trees were only identified to the genus level and these trees were not included in some analyses (see below).

We overlaid the tree point layer with the 2020 Generalized Land Use Inventory polygon dataset (Metropolitan Council, 2020) to categorize the land use type associated with each tree's location. We also overlaid a vector street layer derived from the Minnesota Department of Transportation (Marek‐Spartz, 2023). This layer originally included streets and alleys (all “through” lanes), but we subtracted all alleys because they are generally not managed by municipalities in the same way as streets. We aggregated several of the original 16 land use types to include three land use type categories in our analysis: (1) Street trees (all trees within a 3‐m buffer of streets), (2) recreational parkland (including “recreational parkland” as well as points shown to be located in “water” or “major highways,” as these two land cover types were observed via spot checking to be commonly adjacent to recreational parklands and trees therein were assumed to be parkland trees), and (3) other (all other categories, primarily of the categories “residential” and “unknown”).

**FIGURE 1:**
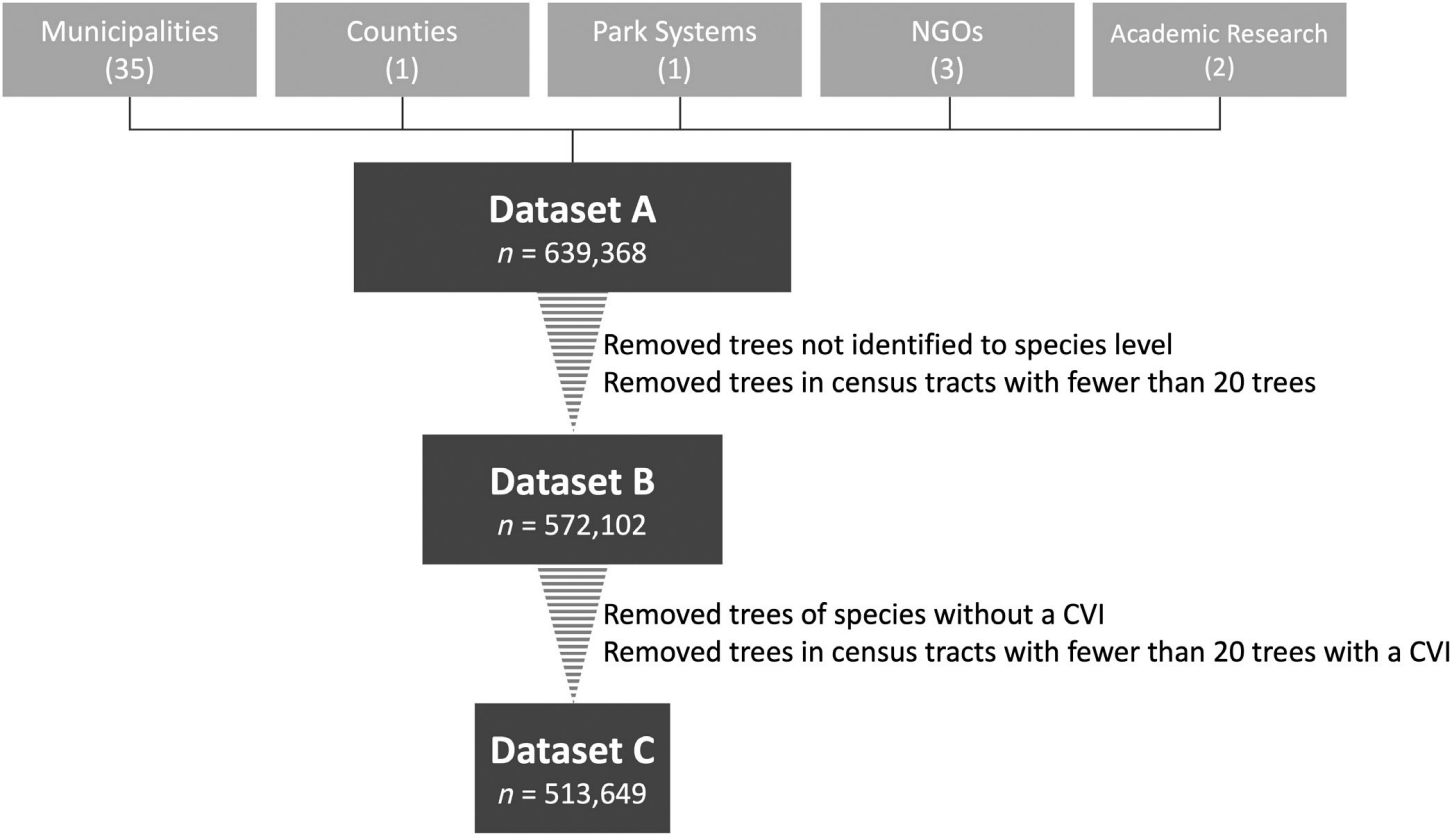
Conceptual figure showing how tree inventories from different organizations were collated and filtered into three hierarchical datasets. The top row of boxes shows the different types of organizations that provided tree inventory data, with the number of organizations of that type shown in parentheses (e.g., data from 35 municipalities were included). Data from all organizations were collated to form dataset A, which was subsequently filtered twice to form datasets B and C as described to the right of each striped triangle. The numbers of individual trees in each dataset (*n*) are shown. CVI, climate vulnerability index.

We used the full database (i.e., dataset A) to analyze patterns at the genus level, but subsequent analyses required sub‐setting the database to account for incomplete data. We calculated tree species composition and taxonomic and phylogenetic diversity for dataset B, which excluded all trees without species‐specific information and trees within any census tract with fewer than 20 data points to avoid analytical issues with small sample sizes. We analyzed climate vulnerability using dataset C, which further excluded all species without a region‐ and species‐specific climate vulnerability index (CVI) and trees within any census tract where fewer than 20 data points fit these criteria (Figure 1).

We quantified both taxonomic and phylogenetic tree species diversity. For taxonomic diversity, we calculated species richness (total number of species per census tract) and Shannon's Diversity Index (*H*). Given that census tracts across the study area varied considerably in land area (mean = 3.03 km^2^, range = 0.21–212.16 km^2^), and more land area provides more potential tree planting sites, we assessed whether species richness scaled with land area. However, we found no positive relationship between census tract land area and species richness of inventoried trees in a census tract (Appendix S1: Figure S1). We acknowledge that species richness is likely confounded by variation in the percentage of total trees inventoried across the study area. The Shannon Diversity Index combines measures of richness and evenness and is calculated as: *H = −∑p*_*i*_ × ln(*p*_*i*_), where *p*_*i*_ is the proportion of the community made up of species *i*.

For phylogenetic diversity, a measure of taxonomic relatedness, we calculated three commonly used metrics that are independent of species richness: Mean Phylogenetic Distance (MPD; Webb, 2000), Phylogenetic Species Variability (PSV), and Phylogenetic Species Evenness (PSE; Helmus et al., 2007). For all phylogenetic metrics, we used the Smith and Brown (2018) phylogeny (https://github.com/FePhyFoFum/big_seed_plant_trees/releases) pruned to match the species in our dataset. Cultivars and hybrids in our dataset were given the genus name and an ambiguous specific epithet (spp.). Species in our dataset missing from Smith and Brown (2018) were substituted with another species in the same genus present in their phylogeny. MPD is calculated as the total phylogenetic distance between all pairs of species normalized by the distance between species in randomized null communities of the same species richness (Webb, 2000). PSV describes phylogenetic variability of species within a community and ranges from 0 (no variability) to 1 (maximum variability). PSE is a modified metric of PSV that incorporates relative species abundances (Helmus et al., 2007). All three metrics showed similar results; for simplicity, we present results in the main text only for MPD and include PSE and PSV results in Appendix S1: Table S1.

We calculated tree taxonomic and phylogenetic diversity for each census tract using the vegan and Picante packages in R (Kembel et al., 2010; Oksanen et al., 2022), and census tracts with fewer than 20 trees were excluded from the analyses to avoid analytical issues with small sample sizes. For each census tract, we also assessed the historic native vegetation type using Marschner's original analysis of Public Land Survey 1847–1907 data (Minnesota DNR, 2022) to determine whether diversity was related to historic vegetation type. Specifically, vegetation classes were aggregated to forest or non‐forest vegetation types and an area‐weighted average of forest versus non‐forest vegetation was calculated for each census tract.

### Tree canopy climate vulnerability

To assess how spatial patterns of tree diversity influence the canopy's vulnerability to climate change, we used a recently published species‐ and region‐specific index of tree vulnerability to climate change (Brandt et al., 2021). For each species, the CVI combines a species' adaptive capacity score (based on literature review and expert analysis) with a zone tolerance score (derived from USDA Hardiness Zone and American Horticultural Society Heat Zone tolerances that were then compared with statistically downscaled climate projections). The CVI assigns species into one of five categories, which we then grouped into three broader categories of climate vulnerability as follows: low (inclusive of low or low‐moderate CVI), moderate (moderate CVI), or high vulnerability (moderate‐high or high CVI). For each census tract, we calculated the proportion of trees in each vulnerability category using dataset C. Phylogenetic signal in climate vulnerability was calculated using Blomberg's *K* (1000 randomizations), comparing the observed *K* value to a white noise null model using the phytools package (Revell, 2012).

### Tree diversity relationships to social vulnerability

To assess the social vulnerability of neighborhoods across the study area, we used the CDC/ATSDR Social Vulnerability Index (SVI), which combines 16 variables from the U.S. Census ACS to identify the relative vulnerability of human communities, particularly in the face of disasters (CDC/ATSDR, 2022). The 16 variables each associate with one of four themes: socioeconomic status, household characteristics, racial and ethnic minority status, and housing type and transportation. The SVI ranks the overall social vulnerability of each census tract against other tracts in each state to calculate a percentile ranking with values ranging from 0 to 1, with higher values indicating greater social vulnerability in a census tract than other tracts in that state. We used the SVI derived from the most recent 5‐year ACS (2016–2020) for the state of Minnesota. The distribution of SVI rankings for all census tracts across MSP was slightly U‐shaped and similar to the distribution of SVI rankings for census tracts included in our analyses (using dataset C). This indicated that our tree inventory data were not biased toward neighborhoods with low or high SVI. In addition to testing relationships between overall SVI and tree diversity, we also examined each of the four individual themes and selected individual variables (i.e., below 150% poverty level, housing cost burden, no high school diploma, and multiunit structures) related to tree diversity.

### Statistical analyses

We performed all analyses in R 4.2.1 (R Core Team, 2022). We used the sf package (Pebesma, 2022) for spatial analysis, and created spatial maps using the tmap package (Tennekes, 2022). We calculated global spatial autocorrelation of each diversity metric, CVI, SVI, theme and variable components of SVI, and median year structure built with Moran's *I* index using an inverse distance decay spatial weights matrix. In contrast to nonspatial correlation indices which uniformly range from −1 to 1, Moran's *I* index varies with the spatial weights matrix, making interpretation of the index challenging. A common rule of thumb follows that a Moran's *I* index greater than 0.3 indicates some autocorrelation, with larger numbers indicating stronger autocorrelation. We used Getis‐Ord (G_*i*_) as a local measure of spatial autocorrelation, which identifies areas of comparatively high associations with neighboring areas. *Z*‐scores for the G_*i*_ statistic are calculated such that large positive values represent clusters of high values of the metric of interest (e.g., hot spot of species richness) and large negative values represent clusters of low values (e.g., cold spot of species richness). Hot spots and cold spots were identified at the 95% CI. We used Welch's *t*‐test to compare metrics of diversity between the urban core and outlying areas and between Minneapolis and St. Paul. We used simple linear regression to examine bivariate relationships between species richness, Shannon's diversity, or phylogenetic diversity and variables related to climate and social vulnerability (because none of our variables showed more than moderate autocorrelation, we did not use spatial autoregressive models).

## RESULTS

### Tree diversity patterns

**FIGURE 2:**
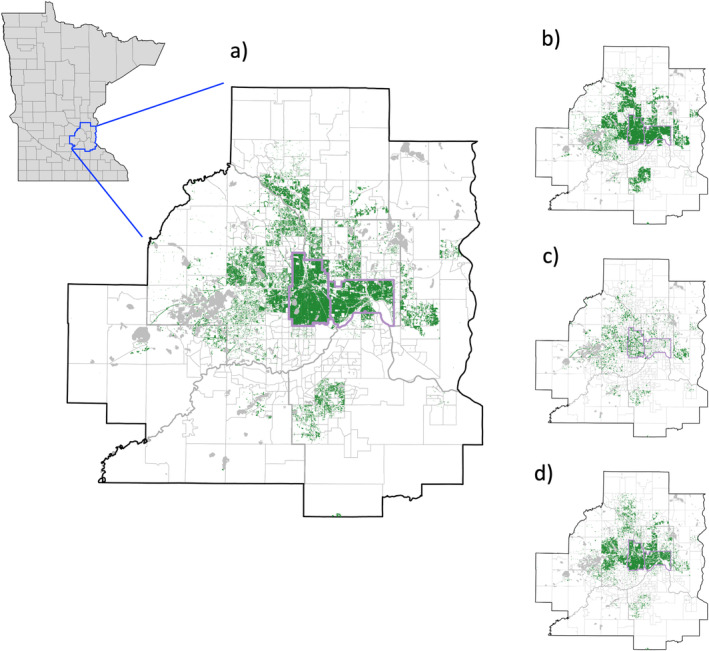
Map of individual trees included in the full tree inventory (dataset A) spanning the seven‐county Minneapolis‐St. Paul Metropolitan Area in southeast Minnesota. All trees, regardless of land use type, are shown as dark green dots in (a). Lighter green dots represent the dataset divided by land use type, specifically street trees (b), recreational parkland (c), and all other inventoried trees (d). The inset in the upper left of (a) shows the study area (outline in blue) with respect to the state of Minnesota (counties delineated with gray lines). For context in the metropolitan area maps, lakes are shown as gray polygons, county lines are delineated with thick gray lines, census tracts are shown with thin gray lines, and Minneapolis and St. Paul are outlined in purple.

Our compiled database of tree inventories across the seven‐county MSP included a total of 639,368 trees spanning 110 cities, townships, or unorganized territories (CTUs, as defined by the U.S. Census) and 648 census tracts (accounting for 83% of all census tracts across MSP). Most (54%) trees in our inventory database were located within Minneapolis and St. Paul (the two largest cities in the study area), with the remaining trees distributed across suburban and exurban areas of the metropolitan area. Two‐thirds (66%) of trees were identified as street trees, with recreational parkland (20%) and residential (10%) accounting for most of the remaining inventoried trees (other land use types included commercial [2%], industrial, agricultural, and vacant [each less than 1%]) (Figure 2). Results generally did not vary across different land use types, and therefore we present results hereafter focusing on all land use types grouped together.

### Tree species composition and diversity

**FIGURE 3:**
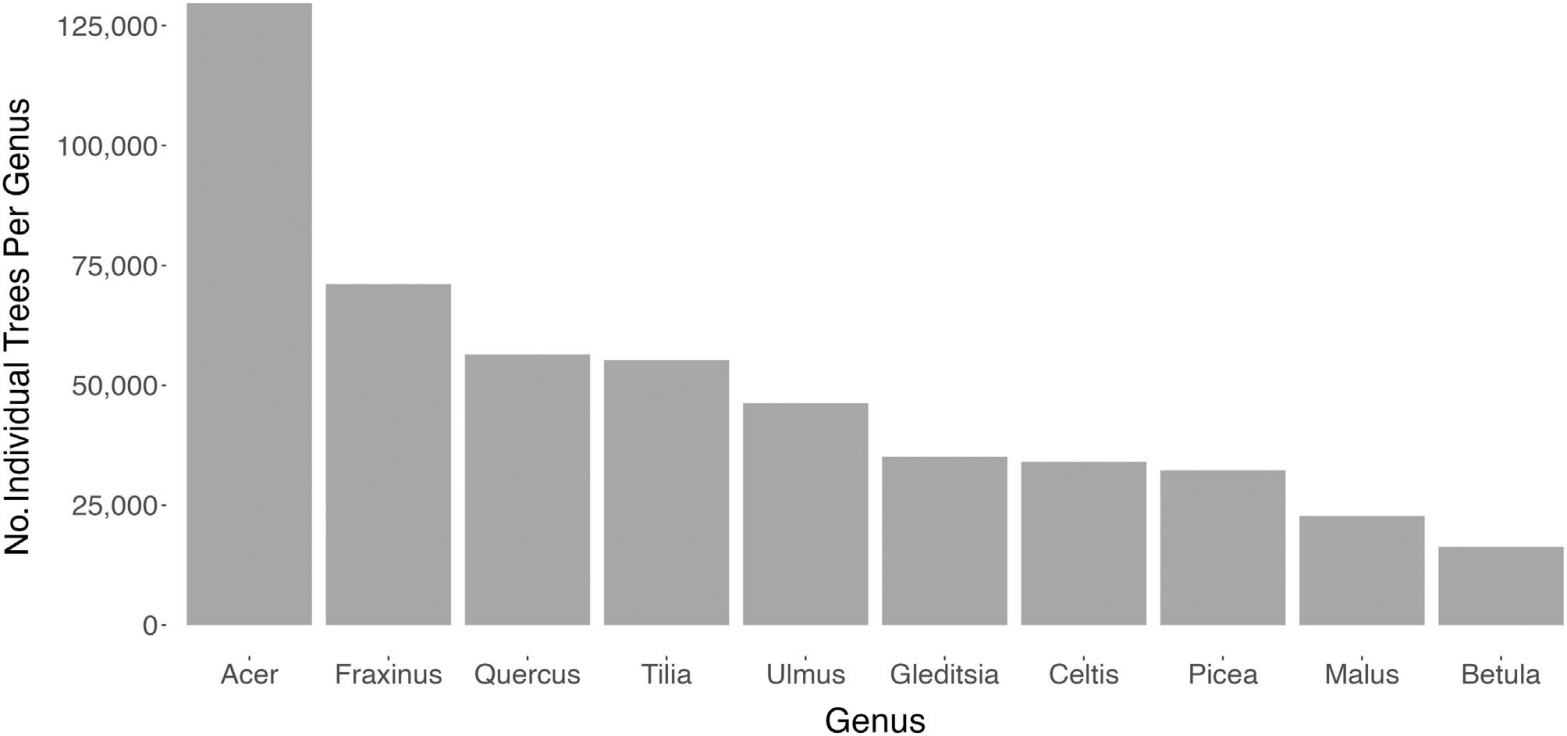
Count of individual trees per genus for the ten most common genera, calculated from the full inventory (dataset A).

In the full database (database A), 101 genera and 449 species were represented. *Acer* was the most common genus (20.3% of all trees) while nearly a third (30.9%) of *Acer* trees were of a single species, *Acer platanoides*. Seven other genera comprised at least 5% of database A, including *Fraxinus*, *Quercus*, *Tilia*, *Ulmus*, *Gleditsia*, *Celtis*, and *Picea* (Figure 3). The phylogenetic tree is presented in Appendix S1: Figure S2. Given recent removals and deaths of *Fraxinus* due to EAB (*Agrilus planipennis*), current prevalence of *Fraxinus* may be overestimated in our database.

**FIGURE 4:**
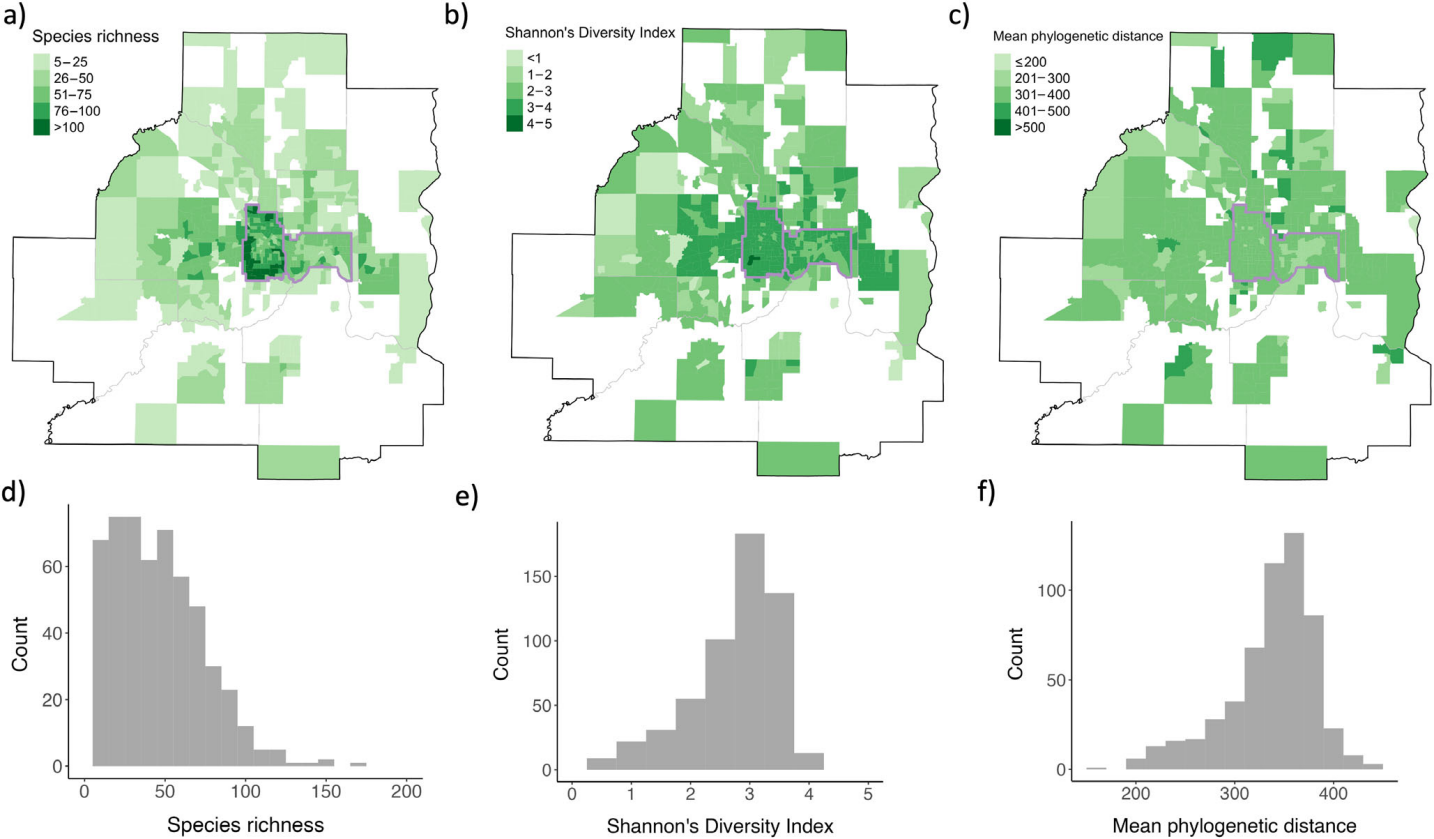
Species diversity calculated for each census tract where sufficient data existed across the 7‐county metropolitan area (using dataset B). Tree species richness (number of tree species) (a), Shannon's Diversity Index (b), and Mean Phylogenetic Distance (MPD) (c) are shown spatially, with darker hues indicating greater diversity. Distributions of tree species richness (d), Shannon's Diversity Index (e), and MPD (f) are also shown. For context, county lines are delineated with thick gray lines, census tracts are shown with thin gray lines, and Minneapolis and St. Paul are outlined in purple.

Taxonomic diversity (i.e., species richness and diversity, calculated from database B and using census tracts as the spatial unit of analysis) varied spatially across the seven‐county study area. Species richness was right‐skewed, while Shannon's Diversity was left‐skewed. Phylogenetic diversity (i.e., MPD) showed less spatial variability and was only slightly right‐skewed (Figure 4). Taxonomic diversity showed moderate spatial autocorrelation (richness: global Moran's *I* index = 0.39, *p* < 0.001; Shannon's Diversity: global Moran's *I* = 0.31, *p* < 0.001), while phylogenetic diversity was not spatially autocorrelated (global Moran's *I* = 0.11, *p* < 0.001). Species richness, Shannon's Diversity, and MPD did not vary depending on the historic (circa 1847–1907) dominance of forest versus non‐forest vegetation.

Mean species richness and Shannon's Diversity across the entire study area were 45 species and 2.73, respectively. Lower taxonomic diversity occurred in suburban and ex‐urban census tracts (species richness = mean 37.45 ± SD 25.5; Shannon's Diversity = 2.53 ± 0.79) compared with the urban core (i.e., tracts within Minneapolis and St. Paul; species richness = 64.41 ± 27.11, Shannon's Diversity = 3.18 ± 0.39; *t*‐test: species richness *t* = 12.41, *p* < 0.001, Shannon's Diversity *t* = 13.57, *p* < 0.001). Phylogenetic diversity (MPD) across the study area was 336.4 (range = 167.1–447.1). There was slightly lower phylogenetic diversity in the urban core than in outlying areas (*t* = −2.65; *p* = 0.008). St. Paul had significantly lower phylogenetic diversity than Minneapolis (St. Paul = 311.73 ± 45.30; Minneapolis = 348.45 ± 24.57; *t* = 7.54, *p* < 0.001). Getis–Ord local G_*i*_ analysis illustrated these local patterns of urban tree diversity (Appendix S1: Figure S3).

### Tree canopy climate vulnerability

**FIGURE 5:**
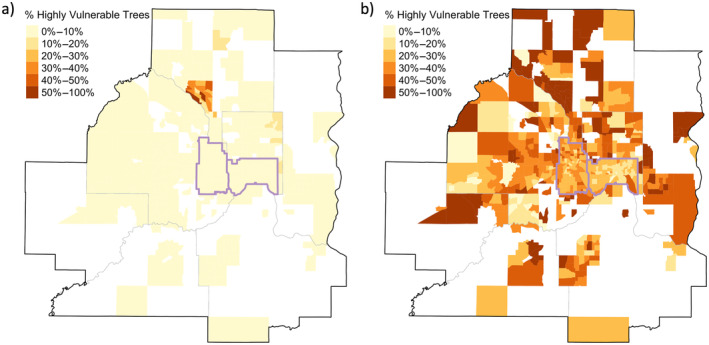
Percentage of inventoried trees that were highly vulnerable to climate change under a low emissions scenario (a) and a high emissions scenario (b). All census tracts with sufficient data (dataset C) are colored (white areas of the map indicate census tracts without inventory data); darker hues indicate a greater percentage of inventoried trees in a census tract were highly vulnerable to climate change. For context, lakes are shown as gray polygons, county lines are delineated with thick gray lines, census tracts are shown with thin gray lines, and Minneapolis and St. Paul are outlined in purple.

Under a low greenhouse gas emissions scenario, the mean proportion of trees across all census tracts with high climate vulnerability within a census tract was 2.5%. Additionally, only 2.6% of census tracts with data (dataset C) had 20% or more of their inventoried trees identified as highly vulnerable, and these census tracts were spatially clumped in one municipality (Coon Rapids) where highly vulnerable *Fraxinus excelsior* (European or common ash) trees were dominant (Figure 5a).

Under a high‐emissions scenario, climate vulnerability was not spatially autocorrelated (global Moran's *I* = 0.12, *p* < 0.001). The mean proportion of trees with high vulnerability within a census tract was 33%. In contrast to the low‐emissions scenario, under high emissions, more than 80% of census tracts with data had 20% or more of their inventoried trees identified as highly vulnerable (Figure 5b; Appendix S1: Figure S4). This change in vulnerability between the low‐ and high‐emissions scenarios was largely driven by a few species that shifted from a low‐to‐high vulnerability rating under increased emissions, namely *Tilia americana*, *Acer saccharum*, and *Acer saccharinum*. For species with a CVI rating (dataset C), 73% were classified as native to North America (non‐natives = 26%, unknown = 1%), with 39% of native trees identified to be of high vulnerability and another 12% to be of moderate vulnerability. In contrast, only 18% of non‐native trees were of high vulnerability, but 57% were moderately vulnerable to climate change (Appendix S1: Figure S5). For a list of climate vulnerability ratings of the 30 most common tree species in dataset C, see Appendix S1: Table S2.

In contrast to our hypothesis, greater taxonomic diversity did not necessarily contribute to lower climate vulnerability by reducing the proportion of climate‐vulnerable trees, as we found no relationship between richness or Shannon's Diversity and the percentage of highly vulnerable trees in a census tract (*p* > 0.05). In contrast to our hypothesis, phylogenetic diversity was positively related to climate vulnerability (MPD: *R* ^2^ = 0.13, *p* < 0.001) such that census tracts with more phylogenetically diverse canopies also had a greater proportion of trees predicted to be highly vulnerable under the high‐emissions scenario. There was no significant phylogenetic signal in climate vulnerability (Bloomberg's *K* = 0.0162, *p* = 0.328).

### Relationship between diversity, social vulnerability, and urban forest vulnerability to climate change

**FIGURE 6:**
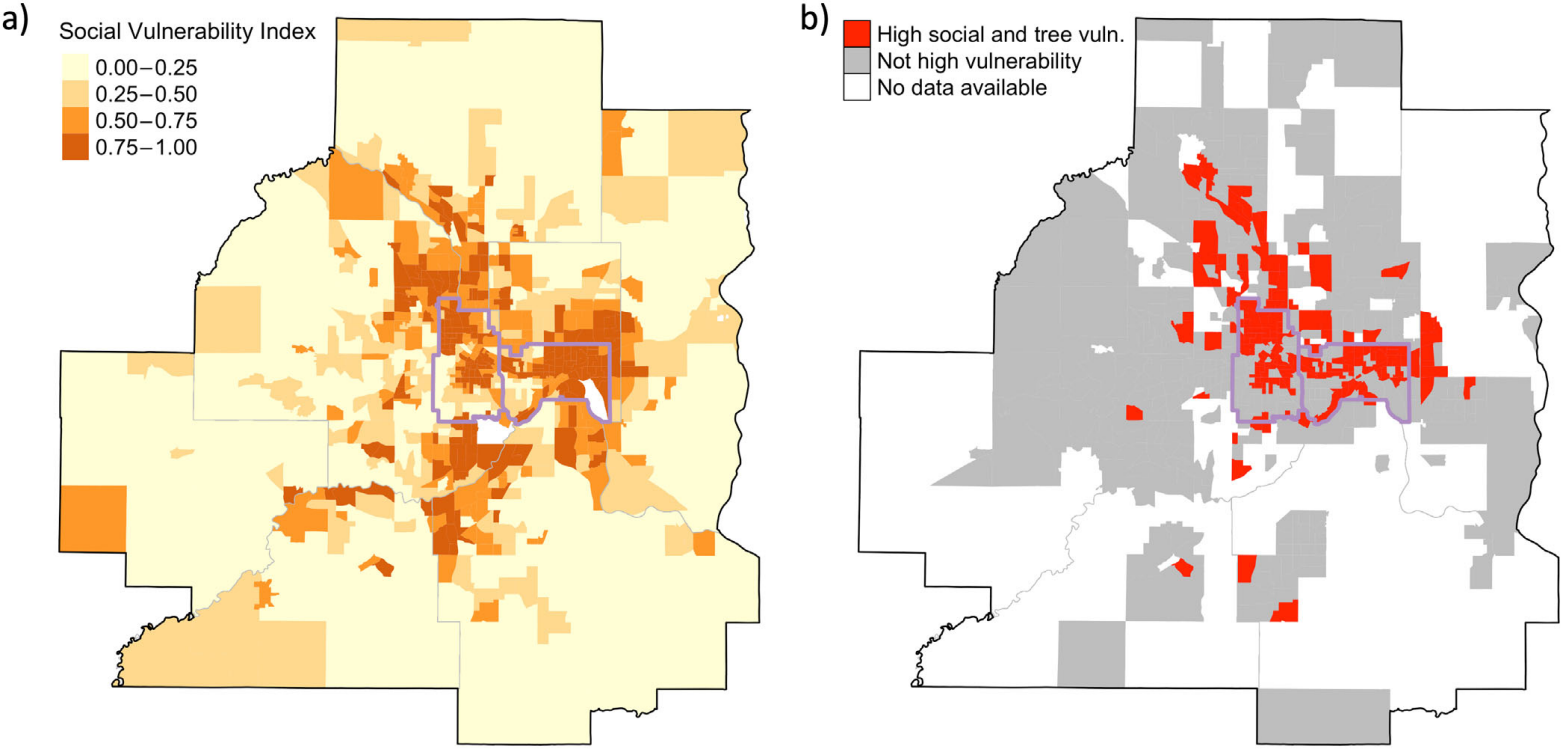
Spatial relationship between social vulnerability and urban forest vulnerability to climate change. The Social Vulnerability Index (SVI) of each census tract (a) is shown with hues of orange, with darker hues indicating greater relative social vulnerability than other census tracts across the state. Areas of both high social and high climate vulnerability (b) are shown, with census tracts highlighted in red where both social vulnerability is above average (SVI > 50%) and more than 50% of inventoried trees are highly or moderately vulnerable to climate change. Census tracts shown in gray have data available for both social and climate vulnerability but are not highly vulnerable for both metrics. Census tracts with insufficient data for climate vulnerability index are shown in white. For context, lakes are shown as gray polygons, county lines are delineated with thick gray lines, census tracts are shown with thin gray lines, and Minneapolis and St. Paul are outlined in purple.

Social vulnerability, as indicated by the CDC/ATSDR SVI, was moderately spatially autocorrelated across the study region (global Moran's *I* = 0.28, *p* < 0.001). Variation in social vulnerability, when assessed with the SVI or with individual themes or variables that make up the SVI, was not predicted by taxonomic or phylogenetic diversity. While the bivariate relationship between diversity and the SVI was statistically significant, very little variation was explained by the predictor variable (species richness: *R* ^2^ = 0.02, *p* = 0.001; Shannon's Diversity: *R* ^2^ = 0.06, *p* < 0.001; MPD: *R* ^2^ = 0.02, *p* = 0.008). However, nearly a quarter (23%) of census tracts with inventory data (dataset C) had both above‐average social vulnerability and a majority of inventoried trees identified to be of moderate or high vulnerability under the high‐emissions scenario (Figure 6).

## DISCUSSION

We compiled a novel, spatially explicit database of urban trees across the seven‐county MSP and showed that urban tree diversity and composition were variable across the study area. More than 25% of the inventoried tree canopy comprised only four species. Although the most common species tended to have low or moderate individual climate vulnerability scores, high prevalence of few species can highlight potential vulnerabilities of the tree canopy to future climate change and disturbances. By spatially overlaying urban forest climate and social vulnerability data, we were able to identify areas where both ecological and social vulnerabilities were high. Our results highlight the roles of both social and ecological factors in shaping urban canopy biodiversity patterns.

### Multiple factors drive patterns of urban tree diversity

Our observational study precludes determining the mechanistic drivers of diversity patterns across MSP. Regardless, we contend that human decision‐making primarily drives observed patterns of MSP urban tree diversity, given that climate and environmental resource gradients are less pronounced across the study area than characteristics of human communities and that humans can alleviate limiting resources such as water and nutrients in urban environments (Hope et al., 2003; Kinzig et al., 2005). Top‐down, municipal‐scale management is apparent, given that census tracts within Minneapolis and St. Paul exhibited greater tree (taxonomic) diversity than outlying municipalities. Such top‐down management interacts with local environmental factors, including disturbances (e.g., storm events and pest and pathogen outbreaks) to influence canopy diversity. For example, the City of Minneapolis has developed a plan to manage for EAB, stating an explicit goal of replanting for enhanced species diversity (Minneapolis Park and Recreation Board, 2023). Bottom‐up management of EAB‐infected ash trees, particularly private and street trees, is also carried out by some residents, although financial burdens imposed by tree removal and replacement limits bottom‐up management of EAB in many cases. Further interdisciplinary research could improve understanding of how ecology, social influences, and governance interact at different spatial scales to drive patterns of urban tree biodiversity (Beninde et al., 2015).

### Managing for urban tree biodiversity in a changing climate

Although most MSP census tracts were dominated by tree species with low‐climate vulnerability under the low‐emissions scenario, the vulnerability of the urban canopy increased dramatically under the high‐emissions scenario. Only one‐third of all species planted across MSP were moderately or highly vulnerable to climate change under the low‐emissions scenario, but two‐thirds of all species were at least moderately vulnerable under the high‐emissions scenario. Using both the low‐ and high‐emissions scenarios in vulnerability assessments can help urban forest managers plan for a range of possible future given the large uncertainties in future technological, socioeconomic, policy, and climatic feedbacks that may influence future greenhouse gas emissions and subsequent climate changes.

Our results emphasize the importance of considering climate adaptation as a part of management actions related to tree biodiversity goals. We observed a positive relationship between tree phylogenetic diversity and the proportion of climate‐vulnerable trees in a census tract (and no relationship between taxonomic diversity and climate vulnerability). The observed higher tree phylogenetic diversity in census tracts with greater urban forest climate vulnerability reflected that high climate vulnerability scores were not restricted to select species or genera. The 10 most common highly vulnerable tree species in the database represented seven plant orders (Fabales, Fagales, Lamiales, Malvales, Pinales, Rosales, and Sapindales) and there was no phylogenetic signal in climate vulnerability due to variation in suitability among closely related species. These results emphasize that increasing diversity alone will not be as effective at reducing climate vulnerability as considering climate change impacts when selecting species for planting. Managing for tree diversity with a focus on future climate‐adapted species increases overall forest resilience and ecosystem functioning, and has also been shown to be positively related to rates of carbon uptake (Belaire et al., 2022).

### Patterns of climate and social vulnerability across the urban forest

We did not find a significant relationship between urban tree diversity and social vulnerability. This contrasts with recent studies in Baltimore, USA, where street tree richness was higher in wealthier neighborhoods (Anderson et al., 2023) and lower in historically redlined neighborhoods (Burghardt et al., 2023). In four cities in eastern Canada, both functional diversity of public trees and canopy cover were lower in more socially vulnerability areas (Landry et al., 2020). Our study included both public and private trees, but most trees in our database were public and formally managed by municipalities. Although our finding of no correlation between tree diversity and social vulnerability does not support our hypothesis, it may reflect that more interacting factors affect the identity of species planted (composition) compared with the presence or absence of a tree (canopy cover). For example, research in Brisbane, Australia, showed that home buyers in neighborhoods of higher socioeconomic status tended to prefer street trees with low tree taxonomic diversity (Plant & Kendal, 2019). More broadly, avoiding disservices (e.g., perceived nuisances) may supersede managing for the benefits of tree diversity (Dickinson & Ramalho, 2022).

Even though social and climate vulnerability did not show a bivariate relationship, some areas of high social vulnerability corresponded with areas of high climate vulnerability. This spatial analysis approach is useful to highlight opportunities for urban forest management to address social and ecological vulnerabilities together. For example, United States Forest Service Urban and Community Forestry (UCF), a covered program under the Federal Justice40 Initiative, requires that 40% of the program's investments benefit disadvantaged communities. Tools such as the Tree Equity Score (https://www.treeequityscore.org/about) have been developed to assess areas of low tree canopy and high social vulnerability, but these tools do not currently include risks to the tree canopy itself. Analyses such as the one presented in this paper can help identify areas for investment in disadvantaged communities that may also be at risk for climate change impacts to the urban tree canopy.

Given the lack of a relationship between tree diversity and social vulnerability, our results show that planting and management of urban tree diversity is not related to neighborhood characteristics of social vulnerability. The highly polycentric governance of MSP creates a multilayered patchwork of urban forest management that contributes to its biodiversity patterns. For example, in any given census tract, residents may manage private lots while also informally managing street trees (e.g., watering during droughts, treating for pests). Formal management of most street trees across a census tract may be under the jurisdiction of a park board or municipality, while some trees along county roads, for example, are managed by the county. Additionally, local community groups may assist with tree planting and maintenance either formally or informally (e.g., The Tree Trust, n.d., www.treetrust.org; Frogtown Green, www.frogtowngreen.com). Tree diversity may have reflected neighborhood characteristics more strongly if our database included more trees in private yards rather than being dominated by public trees. Overall, urban tree diversity is shaped by manifold factors such that the relationship between human community characteristics and tree biodiversity is more complex and less predictable than that with tree cover.

Despite no predictive relationship between urban tree biodiversity and social vulnerability, overlaying urban forest climate vulnerability data with indices of social vulnerability was useful to identify neighborhoods characterized by low social agency due to factors such as socioeconomic status, race or ethnicity, housing type, or mobility, along with low environmental amenities such as urban tree diversity. Neighborhoods that have high social vulnerability may have reduced capacity for bottom‐up management (e.g., fewer financial resources, less connection to community tree planting and maintenance programs), which, in turn, could exacerbate the challenge of maintaining a healthy urban canopy in areas where the tree canopy is highly vulnerable to climate change. This type of spatial analysis exemplifies how urban tree research can inform future investments in urban forest management (Walker et al., 2023).

### Integrating urban forest research and management

Urban forests are increasingly viewed as critical components of healthy and vibrant cities. Maximizing the benefits of urban forests for human and nonhuman communities alike would benefit from thoughtful integration of research and management efforts. First and foremost, accurate and up‐to‐date data on the extent and composition of an urban forest is fundamental to effective management. Our efforts to collate all available data across the seven‐county MSP highlighted the importance of comprehensive urban tree inventories using a standardized format to facilitate integration of multiple inventories across organizations. We recognize it is not reasonable for most cities to inventory every tree within their city limits, but developing and maintaining complete inventories of (at minimum) street trees provides multiple benefits. Municipalities generally have more direct influence on street tree planting and management than other land use types. Nearly all street trees are intentionally planted, and therefore updating inventories can potentially co‐occur with on‐the‐ground management efforts, saving costs on collecting these data. Advances in research can also aid in supplementing incomplete tree inventories, for example through the use of remote sensing tools that allow the characterization of structure and diversity.

To facilitate future integration of tree inventory data, standard formatting of tree inventory data that is user‐friendly to both managers and researchers and readily allows for data harmonization would be helpful. We offer a simple spreadsheet template that organizations can use as a stand‐alone database or can integrate into more advanced databases (Keller, 2024, available for download here: https://doi.org/10.5281/zenodo.13685628). The following standard information for each inventoried tree can help facilitate future analysis and integration across political boundaries: accepted scientific name (https://www.itis.gov/), exact latitude and longitude of tree, land use type, date of data entry, and date of tree removal (if applicable). Other useful data may include species common name, diameter at breast height, signs of disease, and management on tree (e.g., pruning, treatment for disease).

## CONCLUSION

In analyses of a newly compiled inventory of trees across the social‐ecologically diverse seven‐county MSP metropolitan area, urban tree diversity was generally high but spatially variable. These results broadly align with prior research done either over smaller spatial scales or across non‐contiguous cities spanning different climates. Using the large, contiguous MSP study area we were able to control for broad‐scale differences in climate to show that local scale social and environmental factors can influence high spatial variability in the urban tree canopy composition. The vulnerability of the MSP urban forest depends, in part, on the trajectory of greenhouse gas emissions and subsequent climatic changes that play out over the coming decades. U.S. Census variables describing social vulnerability did not correlate with variation in urban tree taxonomic or phylogenetic diversity. However, some areas of high social vulnerability corresponded with areas of high climate vulnerability, highlighting opportunities for urban forest management to address social and ecological vulnerabilities together. Overall, our results emphasize the importance of intentionally managing urban forests for multiple social and ecological co‐benefits.

## CONFLICT OF INTEREST STATEMENT

The authors declare no conflicts of interest.

## Supporting information


Appendix S1:

## Data Availability

Data (Keller et al., 2023) are available in the Environmental Data Initiative's EDI Data Portal at https://doi.org/10.6073/pasta/3d0bd33cd2748a919cb15a57652293ee. Data points from the City of Roseville (entity = “Roseville”; a small subset of the data) are available to qualified researchers by contacting the City of Roseville (Minnesota) Forestry Coordinator (Anita Twaroski; anita.twaroski@cityofroseville.com) and requesting georeferenced and species‐specific tree inventory data from their municipality. Code and the tree inventory template (Keller, 2024) are available in Zenodo at https://doi.org/10.5281/zenodo.13685628.
