# Supplementary material for: Tree diversity across the Minneapolis‐St. Paul Metropolitan Area in relation to climate and social vulnerability
Source: Ecol Appl. 2024 Sep 22;34(8):e3034. doi: 10.1002/eap.3034 (PMC11610676; doi:10.1002/eap.3034)

APPENDIX S1

JOURNAL

Ecological Applications

TITLE

Tree diversity patterns across the Minneapolis-St. Paul Metropolitan Area in relation to climate and social vulnerability

AUTHORS

Adrienne B. Keller, Leslie A. Brandt, Jeannine Cavender-Bares,

Joseph F. Knight, Sarah E. Hobbie

Table S1. Summary of phylogenetic diversity across the MSP study area. Metrics include Mean Phylogenetic Distance (MPD), and Phylogenetic Species Evenness (PSE), Phylogenetic Species Variability (PSV).

Metric	Mean	SD	Minimum	Maximum
MPD	336.6	45.3	167.1	447.1
PSE	0.429	0.104	0.00885	0.655
PSV	0.518	0.0697	0.257	0.688

Table S2. Comparison of climate vulnerability in low vs. high emissions scenario for top 30 most common tree species across the Minneapolis-St. Paul (MSP) metropolitan area that have a climate vulnerability rating (from dataset C). Species are listed in descending order of dominance in dataset C. Climate vulnerability ratings that changed between low and high emissions scenarios are highlighted in red. Native status to North America is also listed.

Tree species	% of dataset C	Climate vulnerability low emissions	Climate vulnerability high emissions	Native status
Fraxinus pennsylvanica	10.4	low	low	native
Acer platanoides	7.8	low	moderate	non-native
Celtis occidentalis	6.6	low	low	native
Tilia americana	5.6	low	high	native
Acer saccharum	4.9	low	high	native
Ulmus americana	4.7	low	low	native
Acer saccharinum	3.5	low	high	native
Tilia cordata	3.4	low	moderate	non-native
Quercus bicolor	3	low	moderate	native
Acer x freemanii	2.8	low	moderate	native
Gymnocladus dioicus	2.7	low	low	native
Betula nigra	2.5	low	low	native
Acer rubrum	2.4	low	low	native
Ginkgo biloba	2.4	low	low	non-native
Quercus macrocarpa	2.2	low	low	native
Picea glauca	2.2	low	high	native
Syringa reticulata	2.1	low	moderate	non-native
Picea pungens	2.1	low	high	native
Quercus rubra	1.9	low	low	native
Fraxinus excelsior	1.3	high	high	non-native
Acer negundo	1.2	low	high	native
Populus deltoides	1.2	moderate	moderate	native
Catalpa speciosa	1.1	low	high	native
Quercus alba	1	moderate	high	native
Quercus ellipsoidalis	1	moderate	high	native
Ulmus pumila	0.9	low	low	non-native
Picea abies	0.9	low	high	non-native
Amelanchier x grandiflora	0.9	low	high	native
Acer ginnala	0.8	low	moderate	non-native

Figure S1. Relationship between census tract land area and species richness using dataset C.

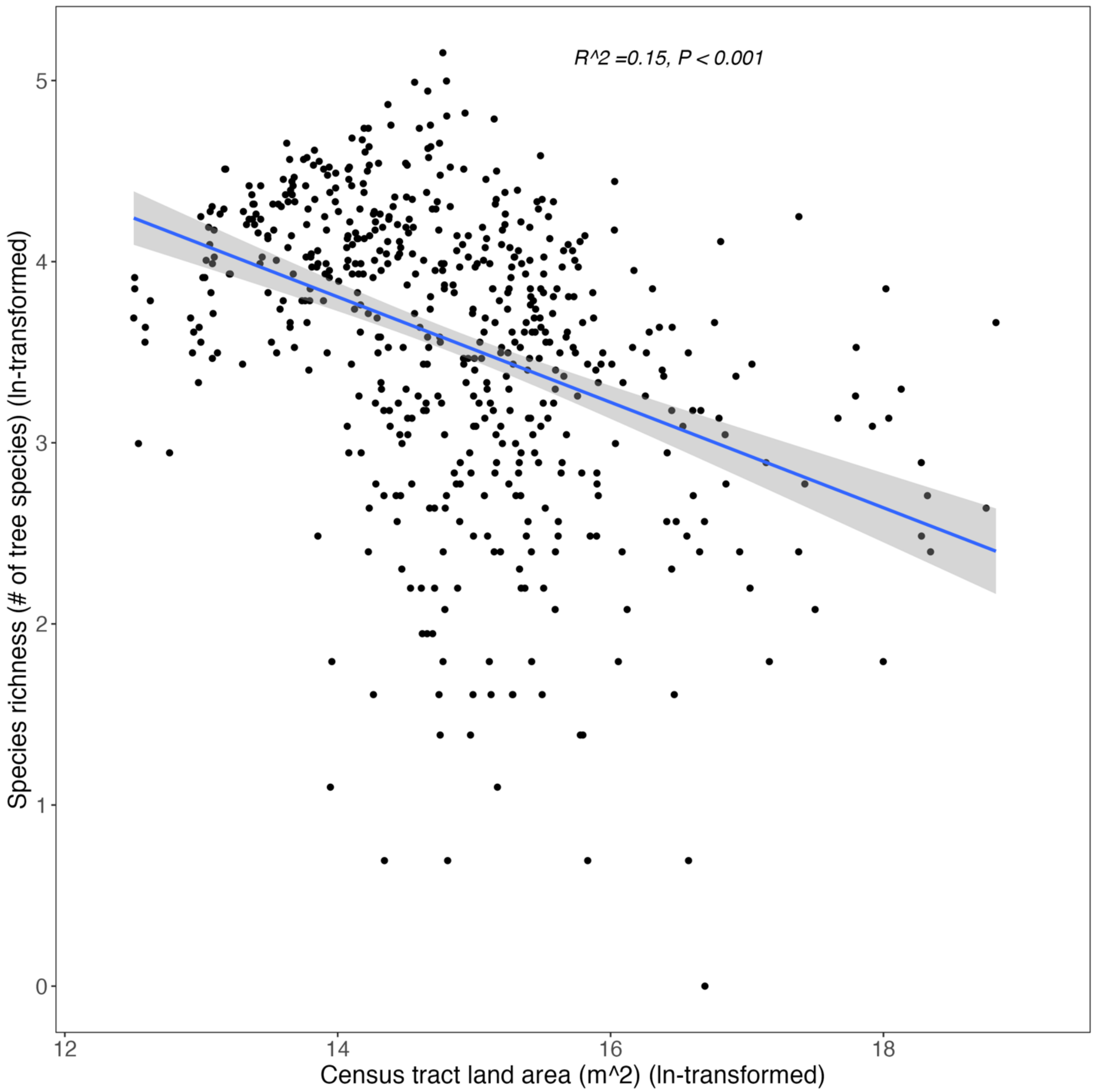

Figure S2. Phylogeny of urban tree species inventories across the Minneapolis-St. Paul (MSP) metropolitan area. Angiosperms colored in red and gymnosperms colored in blue.

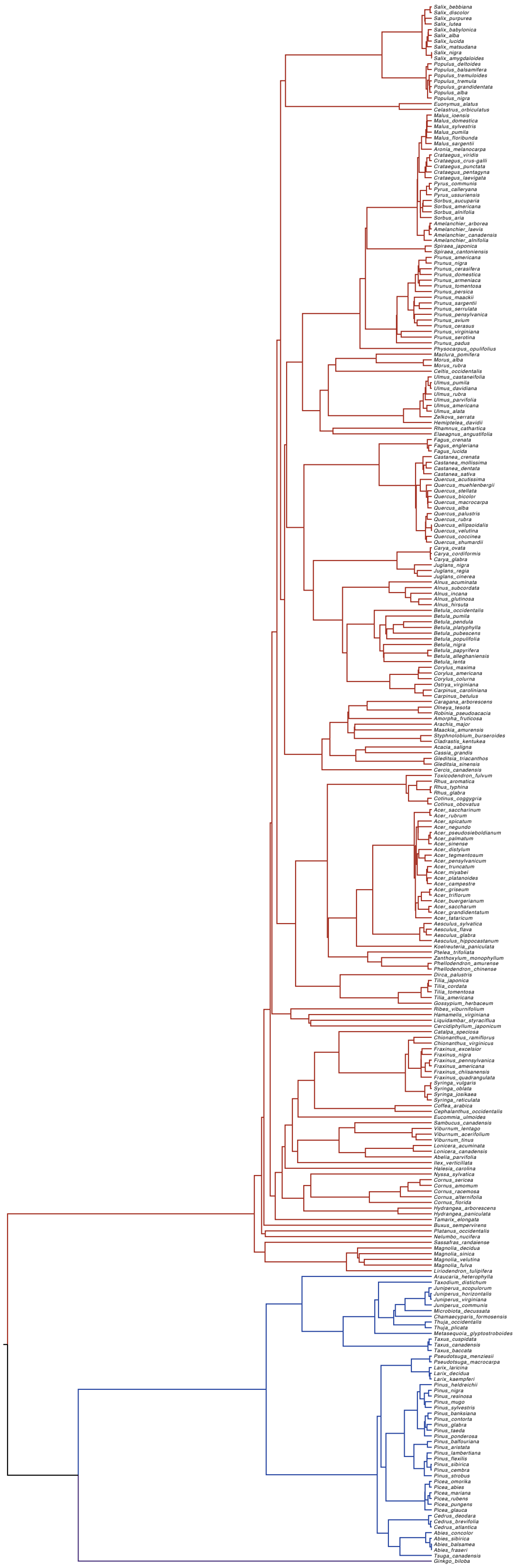

Figure S3. Getis-Ord local G_i spatial patterns of tree species richness (a), Shannon Diversity Index (b), and Mean Phylogenetic Diversity (c). Areas of high local diversity are shown in red and areas of low local diversity are shown in blue. White areas of the map indicate census tracts without inventory data. For context, county lines are delineated with thick gray lines, census tracts are shown with thin gray lines, and Minneapolis and St. Paul are outlined in purple.

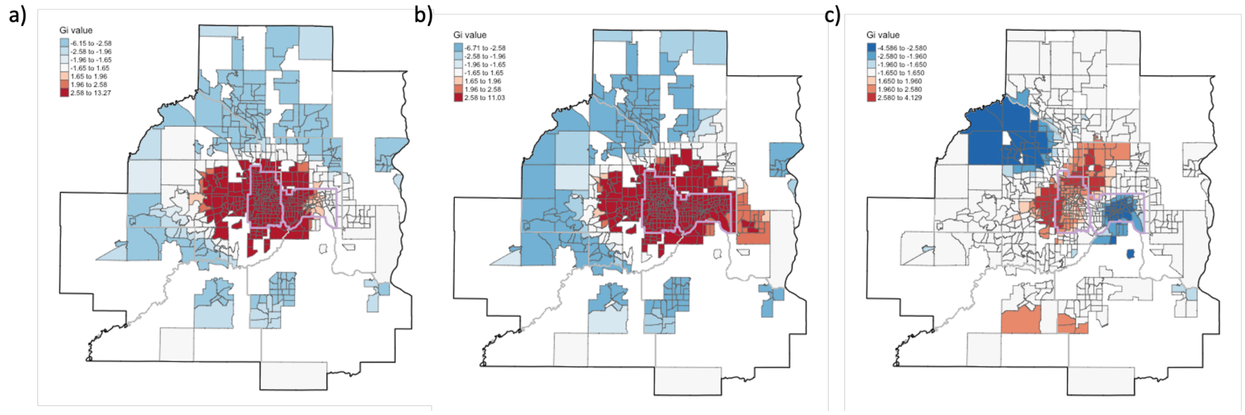

Figure S4. Histogram of percentage of trees in a given census tract that had a) low, b) moderate, or c) high vulnerability to climate change under the high emissions scenario.

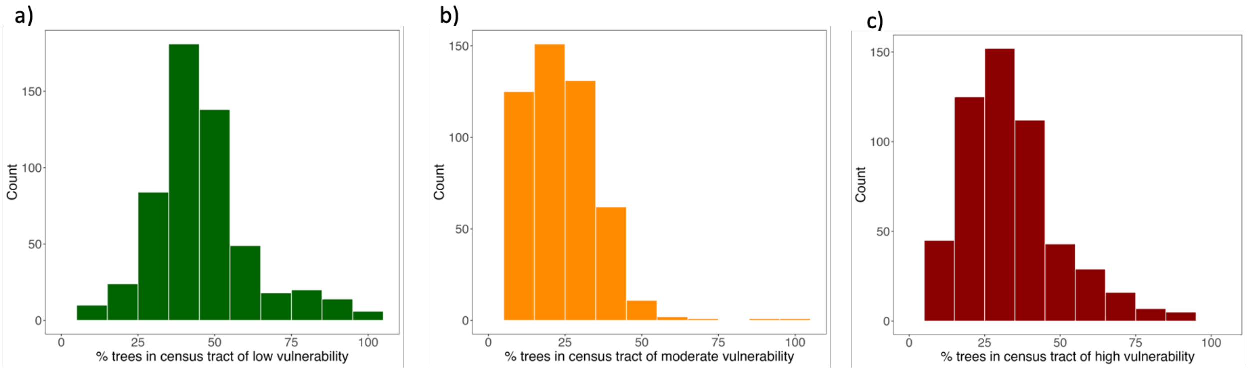

Figure S5. Percentage of a) native and b) non-native inventory trees that were highly vulnerable to climate change under a high emissions scenario. All census tracts with sufficient data (dataset C) are colored (white areas of the map indicate census tracts without inventory data); darker hues indicate a greater percentage of (native, a, or non-native, b) inventoried trees in a census tract were highly vulnerable to climate change. For context, county lines are delineated by grey lines and Minneapolis and St. Paul are outlined in purple.

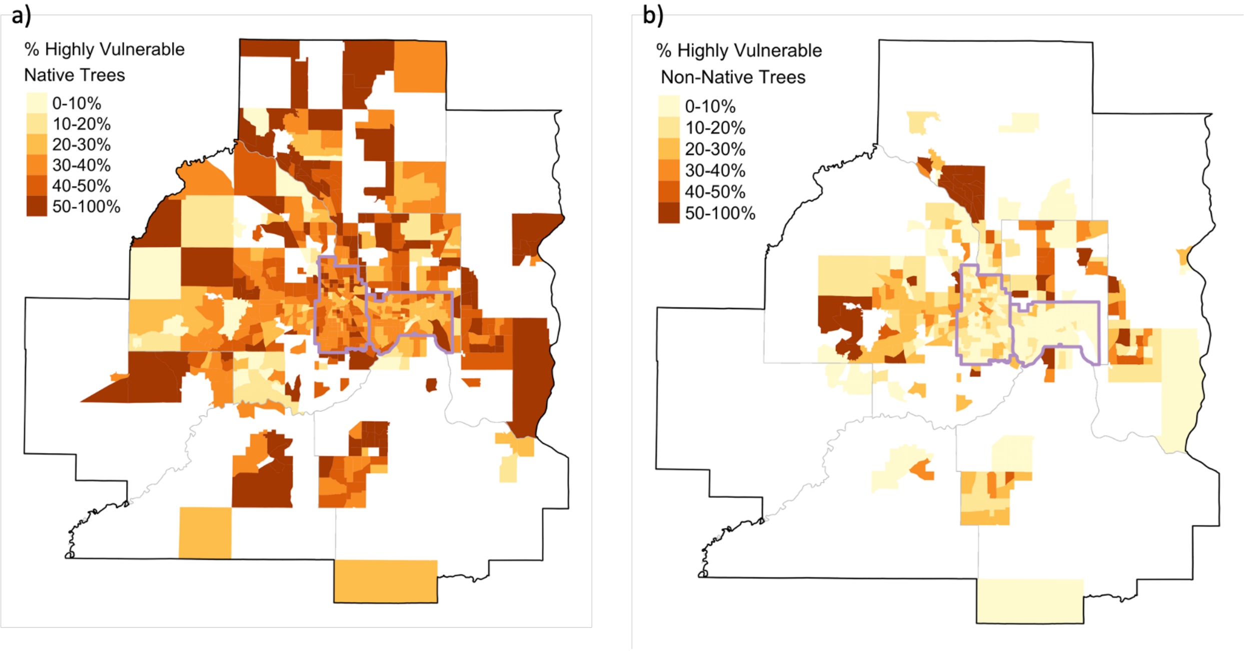

Supplement: Supplementary file 1 — Appendix S1: [file EAP-34-e3034-s001.pdf]
